# Initial evaluation of measurement properties of the Work Environment Impact Questionnaire (WEIQ) - using Rasch analysis

**DOI:** 10.1186/s12955-024-02260-z

**Published:** 2024-05-31

**Authors:** Elin Ekbladh, Moa Yngve, Jeanette Melin

**Affiliations:** 1 Department of Health, Medicine and Caring Sciences, Linköping University, Norrköping, 601 74 Sweden; 2 Department of Women’s and Children’s Health, Uppsala University, Uppsala, Sweden; 3 Division Safety & Transport, Department Measurement Science and Technology, RISE Research Institutes of Sweden, Gothenburg, Sweden

**Keywords:** Construct validity, Measurement, Model of human occupation, Person-environment fit, Work ability, Work environment

## Abstract

**Background:**

To provide both preventive and rehabilitative conditions in a workplace, one must understand how employees experience work demands. Such an understanding can be obtained from each individual with valid and quality-assured questionnaires. The Work Environment Impact Questionnaire (WEIQ) is a new questionnaire for measuring employees’ self-perceived work ability in relation to their specific workplace environment. The purpose of this study was to assess the measurement properties in terms of construct validity of the WEIQ.

**Methods:**

A cross-sectional survey study was conducted with 288 respondents from three different workplaces involving assisted living personnel, vocational rehabilitation personnel and personnel at a research institute. The measurement properties of the WEIQ were assessed according to Rasch Measurement Theory (RMT), including assessment of item-to-sample targeting, threshold ordering, item fit statistics, unidimensionality and reliability.

**Results:**

Item fit, i.e., fit residuals, item characteristic curves (ICC) and chi square values, were all satisfactory, and no disordered thresholds were present after collapsing the lowest response categories. However, issues with local dependent (LD) item correlations was present in 7.6% cases, four items showed statistically significant differential item functioning (DIF), where 11% of the respondents had person fit residuals outside the recommended range of ± 2.5 and the t-test for unidimensionality did not meet the criterion of 5%. Scale-to-sample targeting and reliability (0.92) were good. LD could be resolved with testlets and at the same time maintaining fit and improving dimensionality, but then the reliability decreased to 0.82.

**Conclusions:**

This study provides an initial validation of the WEIQ to be used for assessing employees’ self-perceived work ability. Most measurement properties were acceptable, but further exploration of LD, DIF and unidimensionality in additional work settings and with larger sample sizes is warranted.

**Trial registration:**

Not applicable.

**Supplementary Information:**

The online version contains supplementary material available at 10.1186/s12955-024-02260-z.

## Background

People on sick leave, as well as the length of sick leave, have increased in most European countries [1]. One of the main reasons to go on sick leave is work environment-related problems, such as an overly high workload or prolonged sitting. For instance, in Sweden, almost one-third of the employed population has experienced work environment-related problems, and approximately one-third of them have been absent from work due to those work-related problems [2]. Conditions for remaining at work and returning to work more quickly after sickness absenteeism can then be improved if work environment-related problems are addressed [1, 3]. However, to provide both preventive and rehabilitative conditions in the work setting, one important factor is to understand how employees experience work demands in relation to their capacity and prerequisites [4, 5], i.e., self-perceived work ability.

An individual’s work ability and productivity reach optimal levels when they find satisfaction in their work experience, and there is a harmonious alignment between the work demands, individual abiities, and needs, and vice versa [6]. Further, the perceptions of the work environment are linked to personal factors, including values, interests, thoughts about one’s abiities, and expectations regarding work [7]. Personal factors such as this indicate that health implications could not be assessed from work characteristics per se [4]. This suggests that to bring about improvements in work conditions, employee involvement is needed [5], where the match between workers’ abilities and the demands of their specific work environment needs to be considered rather than just the work conditions themselves [4]. This match between the person and the environment is referred to as *person-environment fit* [7]. The Work Environment Impact Questionnaire (WEIQ) [8] is a new questionnaire for assessing self-perceived work ability (i.e., person-environment fit) within a specific work setting. The WEIQ may be used for preventive purposes to survey how employees perceive their work ability and thereby capture potential conditions that need to be addressed before they become problems, highlighted as an important challenge in order to support people with common mental disorders, as one example [9].

The theoretical underpinnings of the WEIQ stem from the Model of Human Occupation (MOHO) [6] and its accompanying interview assessment, the Work Environment Impact Scale (WEIS) [10, 11]. While the WEIS has been recognized as a practical tool in vocational rehabilitation practice, aiding in the identification of rehabilitation needs and elucidating an individual’s perception of their work environment [12–15], professionals often cite constraints such as limited time and resources, compounded by the time-intensive nature of assessments, as a significant impediment to the use of psychometrically valid tools, observation protocols, and questionnaires [16–18]. The contention is that the scarcity of time might lead professionals to bypass the assessment process in favor of immediate intervention, a choice seldom advantageous for the individual in question [17]. Thus, a descriptive “snapshot” providing a quick overview that guides the subsequent instrument choices for both the assessment process and intervention is valuable [6, 19]. A time-efficient assessment and accompanying adaptation of the work environment can improve the opportunity for the individual to stay in work, return to work more quickly or start vocational rehabilitation, which positively affects the possibility of retaining a job [20, 21]. In line with that, the WEIQ was developed to time-efficiently grasp how individuals percive their work ability within a specific worksetting. The 33 items in the Work Environment Impact Questionnaire (WEIQ) was grounded in the definitions and content of each item in the Work Environment Impact Scale (WEIS) [14], aligning with its theoretical framework, the Model of Human Occupation (MOHO) [11].

While the MOHO provides a solid theoretical framework [6], this enables the necessity of content validity in a questionnaire [22]. Thus, a next step is to evaluate the WEIQ for its measurement properties to provide valid and reliable measurement information (e.g., [23, 24]). To ensure measurement quality with equal measurement units across the continuum, invariance across groups and unidimensionality, the Danish mathematician Georg Rasch developed a model based on the same underlying principles as physical measurements. According to the Rasch model, self-rated data can be evaluated against a measurement model for guiding the construction of stable linear measures from raw data [25] conequently establishing construct validity [26]. Therefore, the purpose of this cross-sectional study was to provide an initial evaluation of the measurement properties in terms of construct validity of the Work Environment Impact Questionnaire (WEIQ) by using Rasch analysis.

## Methods

### Data collection and respondents

The data were collected through convenience sampling in three work settings, representing different kinds of work environments with different work tasks. The data collection was conducted in the following three settings: among assisted living personnel at a public service organisation (group 1, in short, assisted living personnel); vocational rehabilitation personnel at public service health organisations (group 2, in short, rehabilitation personnel); and personnel at a governmental research institute (group 3, in short, researchers). The main work tasks for the assisted living personnel (group 1) are to support the individual in their home with personal hygiene and daily chores such as cleaning, shopping, and cooking. Rehabilitation personnel (group 2) mainly operate within rehabilitation settings, where their primary responsibilities involve assessing work ability and implementing rehabilitative interventions for individuals with injuries or illnesses, aiming to assist them in restoring or sustaining their work ability. The researchers (group 3) were mainly in offices with computer tasks and meetings both in person and online, but could also include external activities.

Group 1 answered the WEIQ by paper and pencil and used a return envelope at their workplace. Group 2 answered the WEIQ either by paper and pencil or online in connection with their participation in a customized course focusing on assessment of work ability, and group 3 answered the WEIQ online after requesting participation via e-mail. All prospective respondents received an information letter with information about the study, explaining that it was voluntary to take part in the study and that their individual answers would be unidentified after completing the WEIQ. All study procedures performed were in accordance with the ethical standards of the 1964 Helsinki declaration and its later amendments. Informed consent was obtained from all individual participants, who completed either the paper-pencil or the online questionnaire after reading the information letter. The persons’ return of the questionnaires was regarded as informed consent, and persons who did not want to participate in the study simply did not answer the WEIQ. No personally identifiable information or code was asked for in the questionnaire, which made the collected data anonymous, i.e., the answers to a questionnaire could not be derived to any person.

In total, WEIQ responses from 288 respondents were received and included, of which 221 (77%) were women. The number of responses from each group were as follows: group 1, *n* = 81 (77% women); group 2, *n* = 125 (88% women); and group 3, *n* = 82 (60% women).

### Measurement

For each of the 33 items in the WEIQ, which assesses self-perceived work ability, a 4-point Likert scale is used. The respondents are asked to rate their degree of satisfaction, where 0 corresponds to the rating *dissatisfied*, 1 corresponds to the rating *partly dissatisfied*, 2 corresponds to *satisfied* and 3 corresponds to the rating of *very satisfied*.

As satisfaction is rated, one might be tempted to think that the coupling person-item attributes are leniency and quality [27]. However, the respondent’s ratings of satisfaction reflect the percived demands in relation to his or her ability, i.e., the person-environment fit. Thus, the latent construct in WEIQ is associated to the persons’ abilities, and corresponding item attribute tasks difficulties.

### Data analysis

The Rasch model enables separate measures for the individual (i.e., the person ability, θ-value) and the item (i.e., the task difficulty, δ-value) on a conjoint interval scale corresponding to the measurement continuum of self-perceived work ability. With the Rasch analysis, one assesses whether requirements for internal validity and for invariance are met by examining the extent to which observed data accord with the expected values defined by the measurement model. Both statistical and graphical tests are used for eventual differences between observed data and expected values [28] and must be considered as an iterative process with the theoretical underpinnings of the construct purported as measured. The analyses of WEIQ were conducted in RUMM2030 and structured around three central questions outlined by Hobart & Cano [23]:

### Is the scale-to-sample targeting adequate for making judgements about the performance of the scale and the measurement of people?

The Rasch analysis provides items and persons hierarchically ordered according to their relative difficulty (i.e., task difficulty, δ-values) and relative ability (i.e., person ability, θ-values) on the same interval continuum of logit. Thus, the item-person value distributions were examined individually as well as in relation to each other, both numerically and graphically. The better the person’s values match the item values, the greater the potential for a precise measurement [23].

### Has a measurement ruler been successfully constructed?

To examine the extent to which observed data accord with the expected values, several tests are required, and these tests are considered in an iterative process. First, in a polytomous scale, monotonicity of items is expected, i.e., the thresholds should be sequentially ordered [29]. If disordered thresholds occur, collapsing categories could solve this [24]. Likewise, collapsing categories can help if there are very few respondents using one response option. Second, item values should provide a meaningful story of what it means going from lower to higher difficulty. As there is no ordinal theory underpinning the items in the WEIQ, the item values were judged according to their clinically logical order and linked to relevant known qualitative aspects. Third, how well the items statistically fit the model was assessed by Fit Residuals, chi-square, and Item characteristic curve (ICC). Ideally, the individual item fit residuals should be between − 2.5 and + 2.5; the chi-square values should not be statistically significant; and the dots of the class intervals should follow the ICC to support good fit [23]. Fourth, local dependency (LD) were evaluated by comparing item fit residual correlations against a relative cut off, that is residual correlations greater than 0.20 above the average correlations indicate local dependency [30]. To handle LD, testlests of sub sets of items were created [31]. Fifth, to ensure invariance across groups, it is crucial that item estimates do not differ between different groups. Thus, tests for Differential Item Functioning (DIF) were statistically evaluated between the three work settings, followed by stepwise item splits and repeated analyses where DIF were present [32]. Due to multiple tests, Bonferroni correction was applied. When DIF was present, this was also assessed in qualitative terms to provide further clinical justification for required splits. Last, unidimensionality was tested according to the Smiths method [33], i.e., the patterning of the first factor in the principal component analysis (PCA) of residuals was used to define two subsets of items, i.e., both positively and negatively correlated items. Subsequently, person ability and θ-values were estimated for each subtest and compared by using an independent *t-test*. To support unidimensionality, it is recommended that the proportion of persons outside *±* 1.96 should not exceed 5%.

### Are the people in the sample is measured successfully?

To assess if the persons are successfully measured item-person distributions, reliability and person fit residuals were evaluated. The mean person values indicate whether the sample is centred or off centred on the items. Skewed person values imply less measurement precision. The person-separation index (PSI) is a reliability indicator where 0 implies all error and 1 implies no error and should ideally be over 0.8 (i.e., corresponding to a separation ratio of *G = 2*) [34], which implies that the measurement uncertainty is not larger than half the object standard deviation [35]. Lower person fit residuals indicate that the respondent’s responses are characterised by low variability, while higher person fit residuals indicate that the respondent’s responses are characterised by irregular high ratings on difficult items and irregular low ratings on easy items. Ideally, for individual reliable assessments, the person fit residual should lie within − 2.5 to + 2.5 [23].

## Results

Below are the results of WEIQ analyses according to RMT, presented for each of the three central questions.

### Is the scale-to-sample targeting adequate for making judgements about the performance of the scale and the measurement of people?

**Fig. 1:**
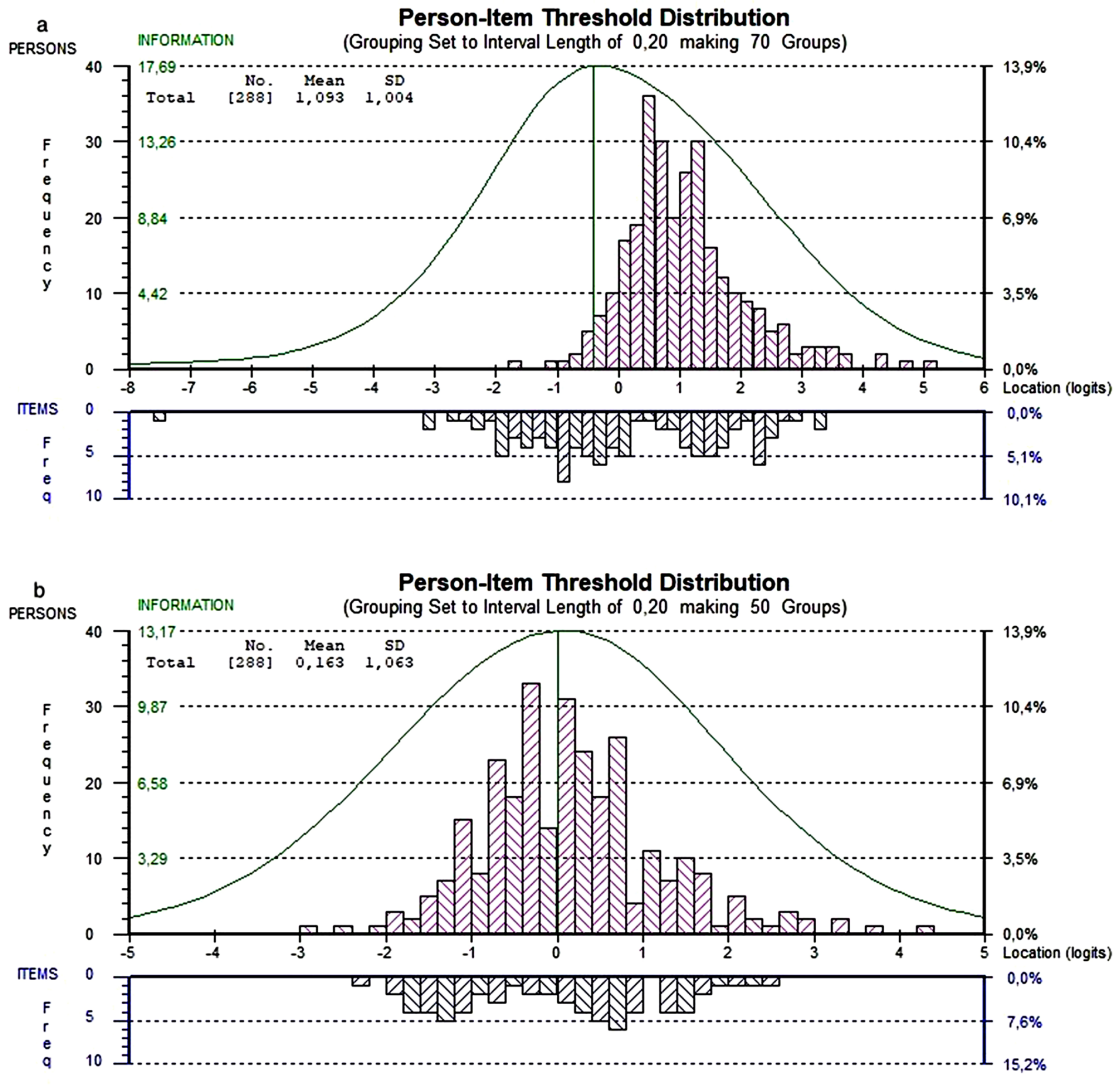
**a & b**: Person item-threshold histograms for WEIQ with **(a)** four response options and **(b)** three response options

The analyses showed a slightly positive skewed person values histogram (Fig. 1a), but there were no problems with disordered thresholds when four response options were used. However, the lowest response option, 0, corresponding to *dissatisfied*, was on average only used by 4% (range 0–22%, median 2%). By collapsing 0 and 1, the distribution among the response options was equalised and at the same time improved the targeting (Fig. 1b); therefore, it was used for the subsequent analysis. Figure 1b shows that all items are covered by the persons and that most persons are covered by the items (mean person value = 0.16, SD = 1.1), indicating great scale-to-sample targeting.

Pink upper bars show person distributions and blue lower bars item threshold distributions scaled on a common logit scale where low values indicate low ability and high values indicate high ability. The green curves show where most information about the persons is provided and are inverse functions of the measurement standard errors.

### Has a measurement ruler been successfully constructed?

After collapsing response options 0 and 1, no disordered thresholds were present, no items showed fit residuals outside the desired range of *±* 2.5, and the ICC dots were close to the curves. Table 1 provides item values, standard errors and fit statistics, and the items are ordered from easiest to the most challenging. The matrix of correlations of item fit residuals is provided in Supplement 1, showing that 7.6% of the pairs of item fit residuals had correlations above the relative cutoff, and that the largest correlations were observed among pairs with similar content, such as item 10 (Collaboration with coworkers) and item 11 (Communication among coworkers) and item 14 (Treatment from manager), and item 15 (Responsiveness from manager). By creating testlets according to the patterns, together with a qualitiave judgement of the item content, fit statistics remained acceptable, and all subsets except one had ordered thresholds. Statistically significant DIF was present for four items: item 16 (Feedback and guidance from manager) for group 1 (assisted living personnel) vs. groups 2 and 3 (rehabilitation personnel and researchers), item 23 (Employment security) item 30 (Availability of personnel spaces) and item 31 (Function and comfort of personnel spaces) for groups 1 and 2 (assisted living and rehabilitation personnel) vs. group 3 (researchers). Individual or stepwise item splits did not significantly change the estimated person values. Statistically significant DIF was also present for testlet 1, which included both item 30 and 31, and testlet 4. Group 3 scored higher than expected for testlet 1 and lower than expected for testlet 4. Furthermore, in the t-test for dimensionality, 11.46% were outside the recommended cutoff *±* 1.96, but with the testlets the percentage decreased to 4.36%.

**Table 1:** The WEIQ item fit statistics

Item	Item short form	Location	2SE	FitResid	ChiSq	Prob
32	Value of ones’ job	-1.12	0.21	-0.40	2.23	0.69
33	Proudness of ones’ job	-0.99	0.21	-0.17	1.55	0.82
8	Work hours	-0.74	0.20	0.82	5.78	0.22
3	Physical requirements in job tasks	-0.70	0.20	-0.74	4.13	0.39
6	Stimulation in job tasks	-0.65	0.20	-0.77	12.05	0.02
13	Social involvement with coworkers	-0.60	0.20	1.76	17.46	0.00
2	Possibility to take breaks	-0.57	0.20	1.14	5.88	0.21
23	Employment security	-0.50	0.20	2.22	9.55	0.05
14	Treatment from manager	-0.39	0.19	1.28	5.14	0.27
10	Collaboration with coworkers	-0.35	0.19	0.45	1.31	0.86
9	Work hours influence on life outside job	-0.27	0.19	1.48	8.78	0.07
20	Expectations for planning of job tasks	-0.27	0.20	-1.45	3.55	0.47
11	Communication among coworkers	-0.20	0.19	1.54	5.48	0.24
18	Expectations of commitment	-0.19	0.23	-2.13	19.20	0.00
7	Job enjoyment	-0.17	0.20	-1.67	8.11	0.09
15	Responsiveness from manager	-0.14	0.19	0.08	4.91	0.30
4	Cognitive requirements in the job tasks	-0.14	0.20	-1.26	9.75	0.04
27	Social atmosphere at job	-0.10	0.19	-0.34	6.12	0.19
21	Interaction with others (recipients)	-0.07	0.21	1.27	1.81	0.77
19	Quality requirements	0.16	0.22	-1.58	12.62	0.01
12	Responsibility sharing among coworkers	0.20	0.19	-0.47	8.04	0.09
5	Emotional requirements in the job tasks	0.29	0.21	-0.95	3.39	0.49
30	Availability of personnel spaces	0.37	0.18	0.75	6.36	0.17
1	Workload in relation to available time	0.40	0.20	1.89	11.94	0.02
28	Availability of work equipment	0.44	0.20	0.48	5.25	0.26
29	Standard and quality of work equipment	0.46	0.20	0.00	3.14	0.54
16	Feedback and guidance from manager	0.46	0.19	1.56	5.80	0.21
17	Expectations of efficiency	0.47	0.22	-1.31	6.81	0.15
31	Function and comfort of personnel spaces	0.59	0.18	0.19	4.52	0.34
26	Physical design of workspaces	0.82	0.20	-0.20	2.39	0.66
25	Sensory qualities	0.93	0.19	0.89	7.51	0.11
24	Balance between job effort and reward	1.11	0.22	-2.15	12.37	0.01
22	Rewards from the employer	1.46	0.22	0.07	2.26	0.69

### Are the people in the sample measured successfully?

There were no persons with extreme person measures, and the PSI was 0.92. However, 23 (8%) respondents had person fit residuals below 2.5, and 10 (3%) respondents had person fit residuals above 2.5. With testlets, there were still no extreme person measures and the PSI was, as expected, reduced to 0.82 and fewer respondents 12 (4%) had person fit residuals below − 2.5 and none had fit residuals above 2.5. Individuals’ work ability from the three different work places was spread out across the continuum, although at the group level, group 1 (mean − 0.46, SD 0.83) showed significantly lower self-perceived work ability than group 2 (mean 0.51, SD 0.96). Corresponding significant group differences were observed with testlets.

## Discussion

This study provides an initial validity of the WEIQ to be used for assessing self-perceived work ability. Item fit statistics were acceptable, but local dependency and the t-test for unidimensionality could not be supported. On the one hand, we have indications for WEIQ items to be used as one scale for assessing a higher ordered work ability. More specifically, a clinically meaningful story was created with items ordered in a logical manner following the same pattern of person-environment fit found in earlier studies on the interview assessment WEIS [36–38], in which the present questionnaire WEIQ has its theoretical underpinnings. Furthermore, the issues related to local depenceny could be resolved with the implementation of testlets while maintaining fit and improving dimensionality. Although the reliability decreased, a PSI of over 0.80, not inflated, is still considered as good for the WEIQ.

The calibrated item hierarchy supported the content validity of the scale. Factors in the work environment that were more difficult, such as item 22 “Reward from the employer” and item 25 “Sensory qualities”, are to a great extent decided outside the workers’ control, while easier items, such as item 32 “Value of ones’ job” and item 33 “Proudness of ones’ job”, are more related to how the person experiences the job per se and may be the reason why the person has chosen their particular job. In this study, participants in different work settings self-rated the items in the WEIQ, and for the previous studies on the WEIS [36–38], the data were therapists’ ratings based on data from interviews with people who had health-related disorders and were on sick leave. As expected it was a similar item hierarchy when comparing the WEIQ and the WEIS, since they both reflect a common theoretical ground in the MOHO and the impact of person-environment fit on self-perceived work ability.

A key in the Rasch model to providing comparable measurements of person abilities is invariance, i.e., the comparison between two stimuli should be independent of which particular individuals were instrumental for the comparison [39]. This is typically assessed by DIF, which can be explained by external information and reflect a clinically expected difference [32, 40]. The work setting for the researchers is characterised by an office, which is very different from both assisted living and rehabilitation personnel meeting people at home or in a clinic. In turn, this can explain the present DIF in both answers regarding “Personnel spaces and function” and “Comfort of personal spaces” (items 30 and 31). Differences between those groups were also shown for “Employment security” (item 23) and can reasonably be considered a consequence of less fixed-termed employments among assisted living and rehabilitation personnel. Furthermore, the difference in feedback and guidance (item 16) for assisted living could be explained by the lack of clarity concerning whom employees at different workplaces consider to be their administrative leader and day-to-day activities leader.

In addition to differences owing to the variety in work settings, as pointed out above, values, interests, and thoughts about our ability and expectations about work also affect our perceptions of the work environment [7]. This self-perceived work ability in a specific work environment could be, on a higher-ordered level of person-environment fit, captured by the WEIQ. The overall person-environment fit to be measured with the WEIQ can be related to Andrich’s rope metaphor [41]. A very thick rope, here overall person-environment fit, can form a unidimensional continuum but comprises components of much finer threads that need to be woven together into a rope thick enough for the purpose at hand. This requires assessments of dimensionality to find the most appropriate statistically well-defined and substantively meaningful constructs [42]. In this study, item-fit statistics and the clinically hierarchy support an unidimensional measure of self-perceived work ability. The measure proves valuable in identifying early preventive or rehabilitation needs related to the overall person-environment fit. This could work well on a group level or for capturing people with high risks, but it may not be suitable to specifically tailor preventive and rehabilitation needs for specific parts where the person-environment fit is out of balance and must be addressed. In such cases, the finer treads of the rope – here potential subdomains of person-environment fit – need to be disentangled.

The present findings must be interpreted with some methodological considerations in mind. First, the collapsing of response options 0 and 1 in the analysis was performed due to the low use of option 0. At least 10 observations per response category are recommended when conducting an evaluation of psychometric properties [43], and this assumption was not met for all of the items. The low use of option 0 in the present sample may represent a high person-environment fit, rather than indicate a need for modifications of the rating scale’s response options. Thus, it may not replicate in another sample involving other work settings or persons with pronounced work-related problems. Concerning the clinical utility of the WEIQ, the four-point rating scale involving two options of dissatisfaction (*dissatisfied* and *partly dissatisfied*) is important for several reasons. On the one hand, they are needed to encourage respondents to identify and highlight aspects in the work environment that are suboptimal with respect to person-environment fit. They are also needed to evaluate the effect of implemented interventions, which do not always have the intended effect but still provide some improvement of the person-environment fit. Therefore, further testing of the WEIQ rating scale is needed in other samples and contexts. Second, women were overrepresented in the study sample (77%). This was not surprising, as two of the three work settings could be considered women-dominated jobs, being found in the public service organisation. Even though the sample size is sufficient to provide a good balance for the statistical interperations of item fit in RUMM2030 [44], it did not allow for DIF analyses involving gender due to the overrepresentation of women. In future studies, it would be desirable to include participants from a greater number and other kinds of work settings and professions, ensuring a sample that enables DIF analyses involving gender, as well as other personal attributes of interest. Moreover, the present sample also leads to implications for generalisability beyond work environments similar to those in this study. The choice of a convenience sample was driven by practical considerations regarding recruitment, as well as concerns about not burdening too many respondents in an initial study. Despite limitations in generalisability and a skewed population, we believe that the current sample has provided important insights on the measurement properties of the WEIQ.

Taken together, although the measurement properties were acceptable for the work settings studied here, it could be further improved and evaluated. For instance, conducting additional exploration of dimensionality, LD and DIF across a broader range of workplaces and professions, along with larger sample sizes, could enhance the measurement quality as well as fit-for-purpose assessment of persons’ self-perceived work ability across various work environments. Specifically, potential subdomains within the WEIQ’s construct of person-environment fit needs to be disentangled. In turn, this can support the validity and measurement quality assurance also for assessing specific aspects of the work environment. Together with the higher-ordered level of person-environment fit, that could enhance the clinical utility of the WEIQ in assessing and identifying preventive and rehabilitation needs in specific aspects of work life.

## Conclusions

The WEIQ was developed to be a time-efficient assessment of persons’ self-perceived work ability in a specific work environment, yielding valuable information about employee for preventive and rehabilitative purposes. This study has provided an initial validation of the WEIQ, overall supporting its construct validity and suggesting its practical applicability in its current form. The measurement properties were acceptable, but could be further improved and evaluated. We also propose to examining potential subdomains within the WEIQ’s construct of person-environment fit to provide valid and quality-assured measure also for assessing specific aspects of the work environment.

### Electronic supplementary material

Below is the link to the electronic supplementary material.

Supplementary Material 1

## Data Availability

The dataset used and/or analysed during the current study is available from the corresponding author upon reasonable request.

## Abbreviations

DIF: Differential Item Functioning
LD: Local Dependency
PSI: Person Separation Index
RMT: Rasch Measurement Theory
WEIQ: Work Environment Impact Questionnaire
